# Protocol for quantifying and modeling light scattering in coral microskeletons

**DOI:** 10.1016/j.xpro.2026.104610

**Published:** 2026-06-02

**Authors:** Netanel Kramer, Steven L. Jacques, Daniel Wangpraseurt

**Affiliations:** 1 Marine Biology Research Division, Scripps Institution of Oceanography, University of California, San Diego, La Jolla, CA, USA; 2 Department of Bioengineering, University of Washington, Seattle, WA, USA

**Keywords:** Biophysics, Environmental sciences, Model Organisms, Structural Biology

## Abstract

Light availability, which affects coral photosynthetic performance and health, is regulated by skeleton optical properties. Here, we present a non-invasive protocol for quantifying and modeling light scattering in coral microskeletons. We describe steps for extracting scattering coefficients and converting them to inherent optical properties using theoretical models. We then detail procedures for modeling Monte Carlo photon transport simulations on 3D coral geometries and calculating fluence distributions across skeleton and water layers. This approach enables high-resolution coral light-propagation characterization for ecological research.

For complete details on the use and execution of this protocol, please refer to Kramer et al.^1^

## Highlights

• Instructions for OCT imaging of coral microskeletons
• Procedures for extracting coral optical properties from OCT scans
• Guidance on converting OCT signals to inherent optical properties
• Steps for Monte Carlo light transport simulations in coral skeletons

## Abstract (editor highlights)

Publisher’s note: Undertaking any experimental protocol requires adherence to local institutional guidelines for laboratory safety and ethics.

## Graphical abstract

**Figure:**
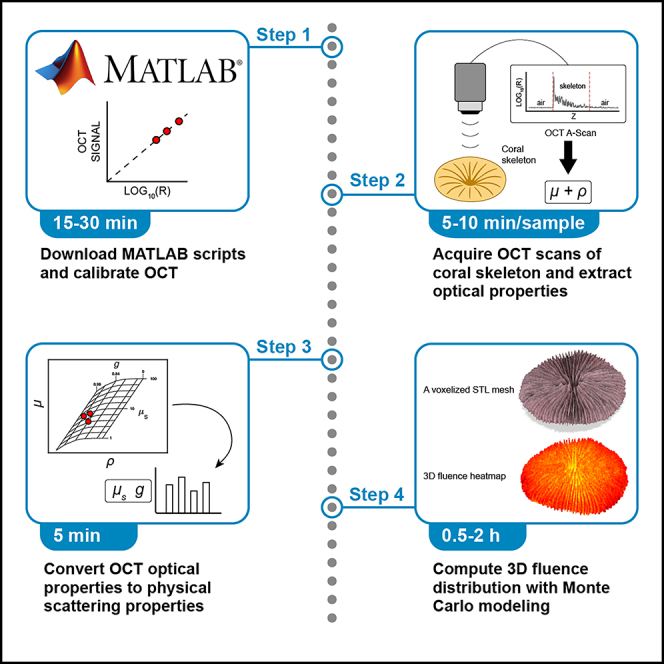

## Teaser

Light availability, which affects coral photosynthetic performance and health, is regulated by skeleton optical properties. Here, we present a non-invasive protocol for quantifying and modeling light scattering in coral microskeletons. We describe steps for extracting scattering coefficients and converting them to inherent properties using theoretical models. We then detail procedures for modeling Monte Carlo photon transport simulations on 3D coral geometries and calculating fluence distributions across skeleton and water layers. This approach enables high-resolution coral light propagation characterization for ecological research.

## Before you begin

Three-dimensional (3D) studies on coral light harvesting provide greater photophysiological insight than simplified 2D models by capturing the complex architecture of coral skeletons.^2^^,^^3^^,^^4^^,^^5^ The workflow described here consists of two key phases for studying the optical properties and light distribution at the coral skeletal microscale.

In the first phase, cross-sectional Optical Coherence Tomography (OCT) scans yield total attenuation coefficients and local reflectance, which are subsequently converted into scattering coefficients (*μ*_*s*_) and anisotropy of scattering (*g*) using theoretical models. The *μ*_*s*_ represents the probability per unit length that a photon will be scattered as it travels through the skeleton (higher values indicate more frequent scattering events), and *g* describes the average direction of scattering events and ranges from −1 to 1, where values close to 1 indicate strongly forward directed scattering. Together, these parameters determine how light spreads through complex structures such as coral skeletons.

In the second phase, Monte Carlo simulations incorporate these optical properties with 3D coral skeleton geometries to model internal fluence rate distributions. The Monte Carlo method simulates the path of millions of individual virtual photons as they interact with the 3D geometry of the skeleton. By calculating where these photons are absorbed or scattered, we can then generate a high-resolution map of fluence, showing the total amount of light available at any given point within the 3D structure. To facilitate learning and validation of the workflow, we provide a lightweight test dataset that allows users to run the full pipeline and verify expected outputs before applying the protocol to their own data. A simple step-by-step visual guide with screenshots is also available in the Zenodo repository (quick_start_guide.pdf) to assist users in setting up and running the pipeline.

This approach was previously applied to investigate the optical contributions of distinct microskeletal features in the coral species *Turbinaria reniformis* from shallow and mesophotic environments,^1^ but is applicable to all scleractinian corals and can be adapted to other calcareous, light-dependent marine species. Here, the coral *Fungia fungites* is used as the demonstration species.

This protocol uses MATLAB R2024a and can be run on any other suitable versions. While 3D models of coral skeletons can be obtained from structure-from-motion photogrammetry or 3D laser scanners for colony-scale bio-optical examinations, we suggest using high-resolution micro-computed tomography (μCT) or OCT systems which have the capacity to capture intricate microscale skeletal features. Lastly, the program is unit-agnostic, meaning it does not enforce any particular units (e.g., cm or mm), and thus, whatever unit is chosen for the voxel size, the same unit should carry through all spatial parameters.

### Innovation

Previous studies of coral light harvesting have often relied on bulk optical measurements^6^^,^^7^ or simplified 2D models^8^^,^^9^ that cannot resolve microscale structural effects on light capture. Foundational studies successfully quantified macro-structural variation in light enhancement and demonstrated their functional importance across many coral species.^6^ While these approaches have provided valuable insights into coral photobiology, the measurements typically average optical effects across entire skeletal elements. As a result, these approaches cannot resolve the fine-scale spatial heterogeneity introduced by specific skeletal features found on the surface of coral skeletons such as individual corallite and coenosteum elements. Furthermore, accurate quantification of light propagation in coral samples remains challenging due to the complex microscale nature of the coral structure.^9^^,^^10^ This protocol integrates high-resolution OCT imaging with voxel-based Monte Carlo simulations to investigate light propagation within coral skeletons at the microscale. OCT provides non-invasive cross-sectional images and 3D renderings of coral skeletons, enabling precise optical and structural characterization without compromising skeletal integrity. The OCT-derived inherent optical properties are then combined with μCT-generated 3D geometries, enabling accurate parameterization of computational Monte Carlo simulations that capture light transport through realistic coral architectures at sub-millimeter resolution. This dual approach bridges structural and optical data, enabling non-invasive quantification of the role of micromorphology in modulating light fields. Lastly, it provides a reproducible framework for linking skeletal microstructure to photobiological function, offering insights into calcareous light-harvesting mechanisms that were previously inaccessible with conventional optical or morphological approaches alone.

## Key resources table

**Table:** 

REAGENT or RESOURCE	SOURCE	IDENTIFIER
**Biological samples**		

*Fungia fungites*	Coral collection from The Steinhardt Museum of Natural History, Tel Aviv	https://smnh.tau.ac.il/en/

**Deposited data**		

Zenodo	https://doi.org/10.5281/zenodo.19055087	https://doi.org/10.5281/zenodo.19055087

**Software and algorithms**		

Dragonfly 2024.1	Comet Technologies Canada Inc	https://dragonfly.comet.tech/
MATLAB R2024a	MathWorks, Inc.	https://www.mathworks.com/
ThorImage®OCT	Thorlabs, Inc.	https://www.thorlabs.com/newgrouppage9.cfm?objectgroup_id=7982
**Chemicals**		
Sodium hypochlorite/NaClO (5% bleach solution)	Thermo Fisher Scientific Inc	https://www.fishersci.com/shop/products/sodium-hypochlorite-5-available-chlorine-solution-reagent-spectrum-chemical/18606586

**Other**		

Micro-Computed Tomography (μCT)	Carl Zeiss Microscopy GmbH	Xradia VersaXRM-510
Optical Coherence Tomography (OCT)	Thorlabs, Inc.	GAN311 Ganymede
DiagPolyTM Plain Polystyrene Nanoparticles, 100 nm	CD bioparticles	DNG-P259
Mineral Oil Light (NF/FCC)	Fisher Chemical™	https://www.fishersci.com/shop/products/mineral-oil-light-nf-fcc-fisher-chemical-3/O1211

## Step-by-step method details

### Coral sample preparation

**Timing: 24–48 h**

Complete removal of organic tissue and pigments is essential for accurate skeletal optical measurements, as residual organic matter can significantly alter light scattering and absorption properties. This preparation ensures that OCT measurements reflect true skeletal optical characteristics rather than tissue-influenced values.

1. Place coral fragments in a well-ventilated fume hood and submerge completely in 5% sodium hypochlorite solution (NaClO). Use a glass or plastic container large enough to allow complete submersion of all coral samples.

**CRITICAL:** Do not use high concentration of NaClO (>10%) as it can damage and erode fine skeletal features, which in turn could affect OCT readings and optical values.

2. Allow samples to bleach for 12–24 h at 25^o^C. Monitor bleaching progress by checking for complete tissue dissolution and loss of coloration.
3. Remove samples from bleach solution and rinse thoroughly with deionized water for 5 min. Gently agitate samples during rinsing to remove loosened organic debris from skeletal crevices.
4. Leave corals to air-dry at 25^o^C for 24 h.

### Downloading MATLAB and codes

**Timing: 5 min**

5. Install MATLAB software from MathWorks: https://www.mathworks.com/products/matlab.html. Ensure you have MATLAB R2024a or later for compatibility with the analysis scripts.
6. Install the following apps from MATLAB: Image Processing Toolbox & Image Processing Toolbox and Computer Vision.
7. Download the zip file containing the OCT and Monte Carlo simulation codes, and OCT image scan examples from the Zenodo link (https://doi.org/10.5281/zenodo.19055087) mentioned in the “key resources table”. Extract all files to a dedicated folder on your computer.

### OCT system calibration

**Timing: 15–30 min**

This section calibrates the OCT system using known reflectance standards and polystyrene microsphere suspensions. Calibration yields the constants needed to convert OCT signal intensity into reflectance (R) and subsequently into attenuation (*μ*) and local reflectivity (*ρ*) values.

***Note:*** The OCT system records a log-encoded signal:

(Equation 1) OCT(z)=a+bln⁡(R(z)),

where *R*(*z*) is the coherent reflectance at depth *z*. For a homogeneous scattering medium:

(Equation 2) $$R\left(z\right)=\rho \;e^{-\mu z}\text{.}$$

Here:

*μ* is the local attenuation coefficient (mm^-1^).

*ρ* is the backscatter reflectivity (dimensionless).

*μ* and *ρ* depend on the inherent optical properties:

(Equation 3) $$\mu =\left(a\left(g\right)\;\mu_{s}+\mu_{a}\right)\;G\text{,}$$

(Equation 4) $$\rho =\mu_{s}\;L\;b\left(g,\alpha \right)\text{,}$$

where *μ*_*s*_ is the scattering coefficient, *g* is scattering anisotropy, *μ*_*a*_ is absorption, *L* is the coherence gate, *α* is the acceptance angle, and *a*(*g*), *b*(*g*,*α*), and *G* are geometric factors defined by the OCT optics. These equations establish the link between the measured OCT signal and the physically meaningful properties later extracted by the *μ*-*ρ* lookup grid.

***Note:*** Materials for calibration: Air/glass, water/glass, and oil/glass interfaces (reference reflectance standards). Deionized (DI) water and mineral oil (Mineral Oil Light (NF/FCC), Fisher Chemical™; *n* = 1.46) were used. OCT system (Thorlabs Ganymede GAN311) with LSM03 objective.

8. OCT settings & preparation.
  a. Set sampling rate to 40 kHz (determined by balancing acquisition speed with signal quality - higher rates reduce noise averaging but increase acquisition time).
  b. Set refractive index to *n* = 1 since scans are performed in air to maximize signal contrast (if sample is submerged in water, use *n* = 1.33).
  c. Use a consistent B-scan field of view. We used 2×2×2 mm.
  d. Determine coherence length (CL) and effective numerical aperture (available from THORLABS GmbH). In this protocol: CL = 5.5 μm and 4.1 μm in air and water, respectively; NA = 0.075.
9. The calibration of the OCT system is accomplished using two sets of standard reflectances:
  a. Reference Standard:
    i. Prepare glass reference standards by taking microscope slides and placing a glass coverslip over the slide (glass/air interface), over deionized water (glass/water interface), and mineral oil (glass/oil interface).
      ***Note:*** During calibration, ensure that the OCT beam is oriented orthogonally to the glass interface (air-glass, water-glass, or oil-glass). Non-normal incidence reduces the effective Fresnel reflectance and introduces systematic calibration errors.
  b. Polystyrene Microsphere (PS) Calibration Standard:
    i. Use 100 nm diameter (*n* = 1.59) in deionized water.
    ii. Create serial dilutions by mixing equal volumes with deionized water: 100%, 50%, 25%, 12.5%, and 6.25% concentrations.
      **CRITICAL:** Ensure reference arm power indicator is in the “green zone” to optimize signal-to-noise ratio and prevent detector saturation.
10. Image Acquisition (please see Table 1 for Recommended OCT acquisition parameters).
  a. Scanning will be performed in the “2D Acquisition” window.
  b. Record background scan with no sample to account for noise artifacts.
  c. Position the glass/air reference under the OCT objective (if sample is submerged in water, begin with glass/water reference).
  d. Monitor the A-scan signal (“Show A-scan”) in real time while adjusting the focus wheel to achieve maximum signal intensity at the reference interface.
  e. For convenience, set maximum intensity at Z = 1 using the mirror.
  f. Click “Export” and save as TIFF file (32-bit) format.
  g. Repeat steps b-f with glass/water and glass/oil interfaces.
  h. Repeat steps b-f with PS.

**Table 1:** Recommended OCT acquisition parameters

Parameter	Recommended value	Acceptable range	Notes
A-scan averaging	20	10 – 30	Higher values reduce noise but increase acquisition time
B-scan averaging	20	10 – 30	Higher values reduce noise but increase acquisition time
Sampling rate	40 (kHz)	20 – 50 (kHz)	Must match system capabilities
B-scan field of view (FOV)	2 x 2 (mm)	1 – 3 (mm)	Keep constant across calibration and sample scans
Refractive index	1.00 (air)/1.33 (water)	N/A	Must match reference interface during calibration
Focal plane placement	Surface peak centered	–	Required for consistent *μ* and *ρ* extraction

**CRITICAL:** Position the reference interface at the same axial depth (within the OCT focus function) used later for coral skeleton measurements to ensure consistent calibration of reflectance and attenuation.

**Figure 1:**
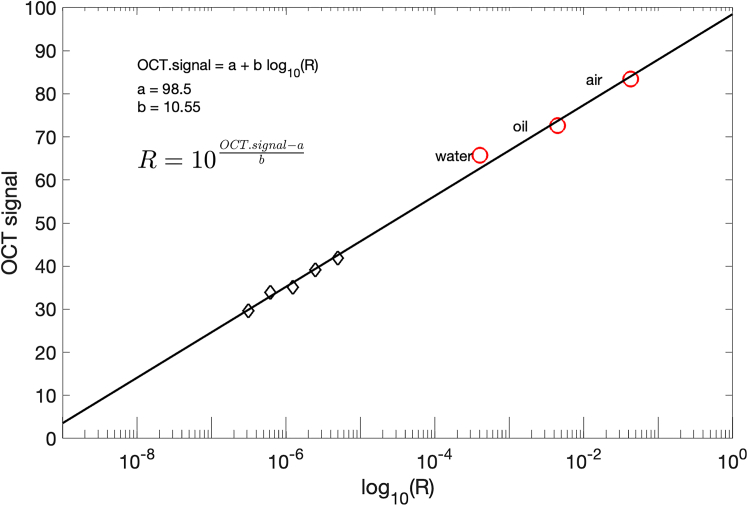
The calibration of the OCT system The expected reflectance is plotted as R on the x-axis. The measured y-intercept of the polystyrene microspheres (black diamond points) and the measured OCT of the air/glass, water/glass, and oil/glass interfaces (red circles) are plotted on the y-axis. The black line through the data serves as a calibration of the OCT system.
  h. The fitted coefficients *a* and *b* are saved.

11. Run the following program:
  >OCT_calibration
  a. You will be prompted to select calibration directory containing TIFF files.
  b. The program will load all TIFF calibration files and calculate B-scan dimensions.
  c. Get resolution of images (microns/pixels). We used res = 2000 / A_rows (2 mm dimension, you would need to change this manually if you have used different dimensions).
  d. The program will extract and analyze A-scan profiles, defining center column and focal plane for A-scan extraction. It will store values and depth at focal plane (iyo). Verify manually and correct focalVal and focalDepth if necessary for nearest peak value and depth.
  e. The theoretical scattering properties (*μ*_*s*_ and *g*) are computed for each PS concentration using Mie theory, then computed predicted *ρ* for each using the following OCT-specific terms:
    >Cstock = 0.025;   % Concentration of stock PS solution[g/ml]
    >dia_um = 0.1;   % Diameter [um] (100 nm)
    >lambda_um = 0.930;  % Wavelength of OCT [um]
    >n_medium = 1.33;  % Refractive index of water
    >n_sphere = 1.59;    % Refractive index of PS
    >PS_density = 1.05;   % PS density [g/cmˆ3]
    >NA = 0.075;   % Numerical aperture of OCT
    >CLum = 5.5;   % coherence length of OCT in air
    >loadWater % loads spectral data for water's optical properties >muaW = interp1(nmWater, muaWater, 10ˆ3∗ lambda_um) / 10;
    >[rhomie, musmie] = calculateMieSphereOCT(Cstock, dia_um,
    lambda_um, n_medium, n_sphere, PS_density, NA, CLum, muaW);
  f. Fresnel reflectance is computed for air/glass, water/glass, and oil/glass using refractive indices:
    (Equation 5) $$R={\left(\frac{n_{1}-n_{2}}{n_{1}+n_{2}}\right)}^{2}\text{,}$$
    >ng = 1.52; % glass reflectence
    >n  = [1 1.46 1.33]'; % air oil water (order recorded)
    >rsp = ((ng-n)./(ng+n)).ˆ2; % Fresnel equation for reflectivity
  g. The calibration curve (Figure 1) is plotted and saved with measured OCT focal-plane signal (y-axis) against known reflectance values (x-axis). Fit:
    (Equation 6) OCTsignal=a+bln⁡(R),

### OCT scanning of coral skeletons

**Timing: 5–10 min (per sample)**

This section describes acquisition of OCT B-scans from coral skeletons after calibration. Proper focus and consistent imaging conditions are essential for reliable extraction of optical properties.

12. Position a cleaned, dry coral skeleton fragment under the OCT objective with the surface of interest facing upward. You may use clay to stabilize irregular fragments.
13. Orient the skeletal surface of interest (e.g., septa, coenosteum, columella, ridges) facing the objective.
14. Using a lab jack, ensure that the coral skeletal surface is positioned at the focal-plane depth used for the calibration reference standards.
15. Record a single B-scan for each region of interest (ROI).
16. Export the B-scan as TIFF format (32-bit) with consistent naming convention indicating the sample’s ID and its area of focus, e.g., sampleID_feature_Bscan01.tiff

***Note:*** The workflow applies identically to both tissue and skeleton samples. However, if calibration was performed in air, scans must be acquired in air. If calibration was performed underwater (typically with live corals), scans must be acquired underwater using the water/glass reference as the focusing standard. Lastly, if scanning underwater, minimize water thickness above the sample, as excess distance attenuates the signal and reduces dynamic range.

### Extraction of optical properties from OCT images

**Timing: 3–5 min (per.TIFF file)**

This section describes the extraction of depth-resolved attenuation coefficient (*μ*) and local reflectivity coefficient (*ρ*) from OCT B-scans using the calibration constants derived earlier.

17. Run optical-property extraction script.
  >OCT_coral_area
  a. Load the calibration file (calibration_constants.mat) when prompted.
  b. Decide on number of slopes for signal fitting (default: num_slope = 1)
  c. Choose a TIFF file from your working directory.
  d. The image will appear with a crosshair. Click twice to define a horizontal ROI (left and right boundaries).
  e. For each A-scan within the ROI, the script:
    i. Extracts the A-scan signal.
    ii. Calibrates reflectance using the fitted a and b.
    iii. Identifies: surface peak, noise floor, exponential decay region, fits one or more exponential slopes to estimate *μ*, and computes *ρ* from the intercept
      ***Note:*** The number of slopes should be determined by the coral’s actual optical heterogeneity. It is recommended to visually inspect the A-scan signal to determine how many distinct decay regions exist in your sample. The optimal fit should capture clear transitions in signal behavior without fitting noise. Typically, one slope for most coral skeletons, or two slopes if a high-scattering superficial layer is visually obvious.
  f. After completing the ROI, the script asks whether to select another ROI. Choose “Yes” to analyze more regions on the same image, or “No” to move to a new file.
    ***Note:*** Each figure (e.g., Figure 2) is saved in the folder “OCT Optical Analysis”. Then, a white dashed line is added to the OCT B-scan image showing which vertical lines have been processed. When finished, all the collected data is compiled into a table, filtered for focus quality (±150 μm from focal plane), and saved as a CSV file for further analysis. Lastly, a.png overlay showing all processed A-scans on the original B-scan is saved. All figures are saved in an automatically created folder: “OCT_Optical_Analysis”.
    **Figure 2:**
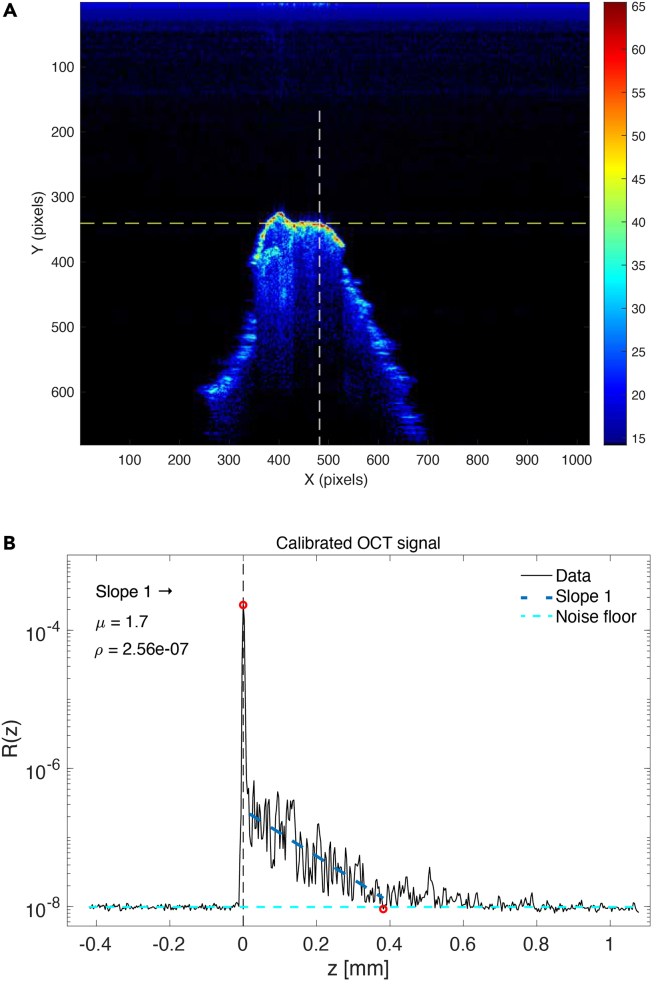
OCT B-scan cross-sectional image (2×2 mm) showing the skeleton structure of a primary septa in the coral *Fungia fungites* and its calibrated OCT A-scan showing optical property extraction (A) Color scale indicates signal intensity (arbitrary units). The white dashed line marks the focal plane position for optimal signal acquisition. Users click twice horizontally to define the region of interest for A-scan extraction. (B) Reflectance vs. depth profile with surface peak (z = 0), exponential decay fit (blue line), and noise floor (cyan line). Red circles mark the surface peak and signal-noise intersection defining penetration depth.

### Conversion of OCT optical properties to physical scattering properties

**Timing: 2 min**

After measuring and extracting the optical parameters from OCT images (attenuation coefficient (*μ*) and backscatter coefficient (*ρ*)), we now convert them into the underlying physical optical properties: scattering coefficient (*μ*_*s*_) and scattering anisotropy factor (*g*). The conversion uses a pre-generated lookup grid based on Monte Carlo simulations that accounts for OCT-specific parameters (numerical aperture and coherence length).

**Figure 3:**
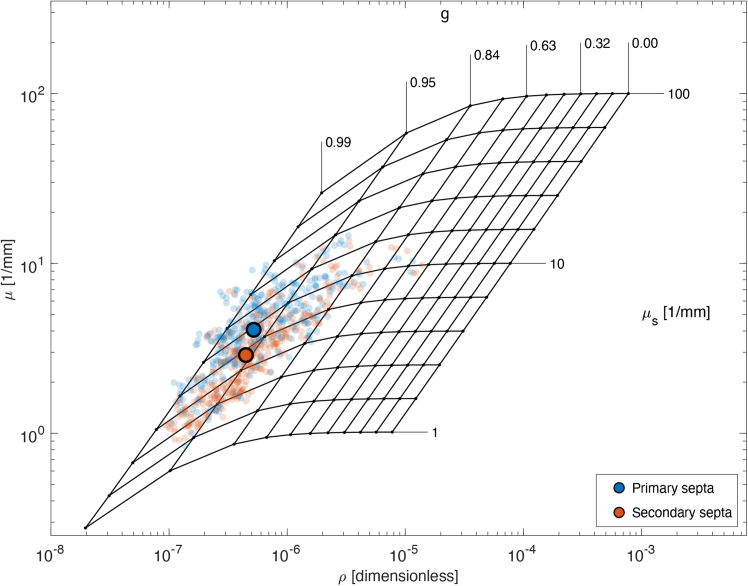
μ-ρ Lookup Grid for OCT-Derived Optical Properties OCT optical property grid showing the relationship between measured properties (*μ*, *ρ*) and physical properties (*μ*_*s*_, *g*). Experimental data points are overlaid and color-coded by area.

18. Run program to convert *μ* and *ρ* values to *μ*_*s*_ and *g*:
  >generate_murhogrid
  a. When prompted, select:
    i. The calibration file (calibration_constants.mat).
    ii. The CSV file generated from OCT optical property extraction.
      ***Note:*** The code generates a theoretical *μ*-*ρ* grid spanning: *μ*_*s*_ = 1 to 100 mm^-1^ and *g*: 0 to 0.98. The grid incorporates OCT-specific parameters (NA, and CL) and was generated by *μ*_*a*_ = 0.0173 mm^-1^ (absorption coefficient of water at 930 nm), a valid approximation for dry coral skeletons at near-infrared wavelengths where scattering strongly dominates absorption.^9^^,^^10^ The experimental data points (*μ*, *ρ*) are plotted on the grid, color-coded by sample group (Figure 3). The script uses 2D interpolation to determine the corresponding *μ*_*s*_ and *g* values for each experimental measurement by finding its position within the theoretical grid. Results are compiled into a table and saved.
      ***Note:*** Optical measurements from coral skeletons exhibit high variability even within single samples due to microscale heterogeneity in skeletal architecture. Some measurements may fall outside the theoretical grid boundaries, indicating physically impossible combinations of *μ* and *ρ* due to noise, calibration issues, or poor exponential curve fits, resulting in NaN values during interpolation. We recommend filtering these out in addition to any outliers and using median values rather than means for robust statistical summarization.

### Monte Carlo light transport simulation

**Timing: 30–120 min (depending on geometry size and voxel resolution)**

The following last phase converts a 3D coral geometry (STL) into a voxelized optical model, launches a Monte-Carlo photon transport simulation, and produces numerical and graphical summaries of the simulated fluence distribution on coral skeleton. The implementation of this script is based on that of mcxyzn.^11^

19. Obtain a 3D model of the coral skeleton using μCT or OCT 3D scanning.
20. Ensure the file name contains no spaces or special characters.
21. Fix the model if necessary to fill holes and remove overlaps and non-manifold edges, then save as a binary STL file.

**CRITICAL:** Verify unit consistency between your STL file and scattering coefficients before running the simulation. The Monte Carlo code expects STL dimensions and scattering coefficients to be the same unit. For example, if your STL file is in millimeters but scattering coefficient in mm, you must either: (1) convert the STL file to centimeters, or (2) multiply the scattering coefficient by 10 (e.g., *μ*_*s*_ = 5 mm^-1^ to *μ*_*s*_ = 50 cm^-1^). Unit mismatches will produce physically incorrect fluence distributions and light penetration depths that are off by orders of magnitude.

**Figure 4:**
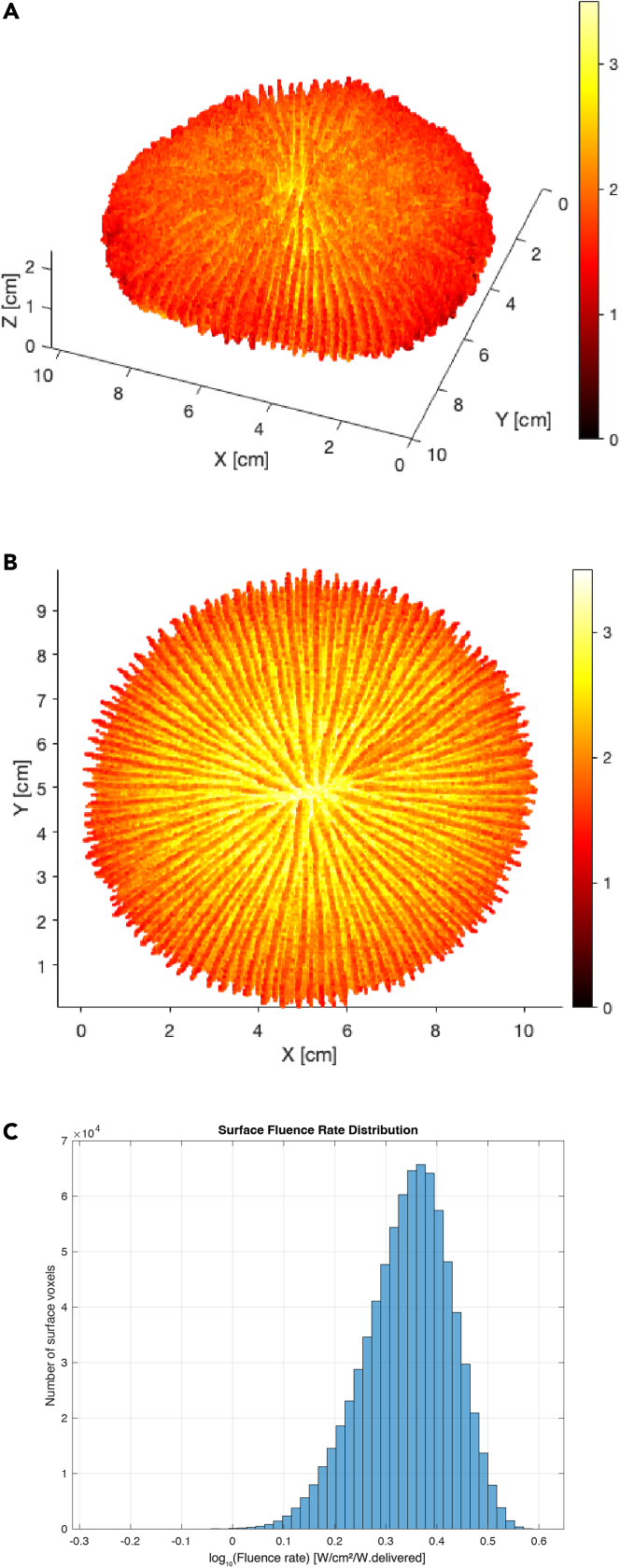
Simulated light distribution in coral skeleton (A) 3D surface fluence visualization showing spatially heterogeneous light fields (0 to 3.5 W/cm^2^/W delivered) across skeletal geometry. (B) Top-view projection revealing light enhancement patterns created by septal architecture. (C) Histogram of surface fluence rates showing median of 2.5× light amplification with distribution reflecting microscale skeletal heterogeneity (n = 1.74×10^6^ surface voxels).

22. Run the photon transport simulation:
  > MC3D
  a. Set simulation parameters and settings: geometry resolution (in pixels), water layer thickness (in pixels), whether to split coral into two optical layers and the split height, and run time of the simulation (in minutes).
  b. Assign optical properties (*μ*_*a*_, *μ*_*s*_, *g*, *n*) for each material layer. For the skeletal layer, use the median values of *μ*_*s*_ and *g*, *μ*_*a*_ = 0.001 mm^-1^ (dry aragonite at 930 nm) and *n* = 1.65.
  c. Select an STL file.
    ***Note:*** The program voxelizes the STL file using the VOXELISE.m that converts it into a 3D binary voxel grid, computes physical extents, and chooses voxel grid dimensions that preserve aspect ratio. Outputs: 3D labeled matrix (1 = water, 2 = coral top, 3 = coral bottom if used), voxel size (cm per voxel), and bounding box dimensions.
    ***Note:*** Monte Carlo setup: The script constructs the optical property table (per material: *μ*_*a*_, *μ*_*s*_, *g*, *n*) and writes a header file (∗_H.mci) listing simulation parameters (Nx, Ny, Nz, voxel size, source/focus, launch/boundary flags, radius/waist, number of materials and per-material optical coefficients). It also writes the binary label file (∗_T.bin).
    ***Note:*** Other launch configurations include Gaussian beam (mcflag = 1), isotropic point source (mcflag = 2), or rectangular beam (mcflag = 3).
    ***Note:*** The simulation creates a destination folder named with the format: [filename]_[simulation time]_[date] containing all outputs.
    ***Note:*** The simulation is executed by calling mcxyzn with the simulation name. Execution status and elapsed time are monitored. On success, output files (∗_F.bin, _T.bin, _H.mci, _props.m) are moved to the destination folder.
    ***Note:*** Very large STL files or fine voxel sizes can exceed RAM limits. Reduce voxel resolution or simplify mesh if simulation crashes.
    ***Note:*** The Monte Carlo output (∗_F.bin) is read and reshaped to the size of the 3D voxel grid. Fluence is normalized by incident irradiance using the calculated beam area and an inverted version for display.
    ***Note:*** The program identifies surface voxels via nearest-neighbor checks (surface voxels have at least one non-coral neighbor).
    ***Note:*** Key metrics computed: median, mean, and 95th percentile of surface fluence, overall amplification (fluences expressed as × incident), and NaN checks.
    ***Note:*** Visualizations (Figure 4): the script generates histograms for coral surface and all layers (the user can choose and extract any layer of interest (e.g., the overlying water (water_mask) or the full skeleton volume (coral_mask)) and 3D scatter of coral surface voxels colored by fluence (top or angled view). 2D top-view scatter and 2D cross-section image are also optional.
    ***Note:*** Saving outputs: a plain-text summary file records geometry, simulation parameters, optical properties, voxel counts, fluence statistics and names of output files. Intermediate CSVs with per-voxel fluence for selected layers are written.

## Expected outcomes

Completing this protocol produces a reproducible, quantitative description of light propagation through coral microskeletons and a set of analysis products suitable for downstream ecological and photobiological studies. At the experiment level, users will obtain calibrated OCT-derived axial profiles and depth-resolved optical properties (attenuation coefficient [*μ*] and local reflectivity [*ρ*]) for each scanned region (Figure 2), together with conversion to inherent scattering metrics (*μ*_*s*_, *g*) via the provided lookup-grid procedure (Figure 3). The optical properties obtained from this protocol can also be combined with independent reflectance measurements to further estimate absorption properties and investigate coral light-harvesting strategies. Increased scattering within coral skeletons and overlying tissues has been shown to enhance internal light trapping, potentially improving absorption efficiency in low-light environments.^1^^,^^12^^,^^13^ Such analyses, however, require additional modeling assumptions that are beyond the scope of this protocol. At the modeling level, users will obtain voxelized coral geometries, Monte Carlo-simulated 3D fluence volumes (Figure 4), and summary statistics (median, mean, 95th percentile) for surface and bulk fluence that link skeletal microarchitecture to local light availability.

In this protocol, *F. fungites* is used as an example species to demonstrate the workflow and expected outputs. However, the OCT calibration, lookup-grid inversion, and Monte Carlo simulation framework are species-agnostic and do not require modification for application to other scleractinian coral skeletons. Consequently, the optical parameters reported here for *F. fungites* should be interpreted as illustrative reference values rather than fixed parameters to be reused across species. Instead, users should expect species-specific variation in the retrieved scattering coefficient (*μ*_*s*_) and anisotropy factor (*g*), reflecting differences in skeletal microstructure, porosity, and surface morphology. Scattering coefficients for coral skeletons typically range from approximately 2.5 mm^-1^ to 60 mm^-1^ across different species, though individual measurements may vary based on skeletal microstructure and measurement location.^1^ For anisotropy factors, coral skeletons are mostly forward scattering with values of 0.8 to 0.99. Moreover, our results showed lower scattering in the secondary lower septa compared to the primary septa of *F. fungites*, consistent with patterns observed in species in which lower ridges, grooves, and intercostal troughs typically exhibit reduced scattering. However, this relationship may vary among species and has yet to be fully characterized.

Moderate variation in the retrieved optical parameters (±10% in *μ*_*s*_ and ±2% in *g*) produced only modest changes in the simulated fluence distribution (Table 2). This sensitivity analysis revealed that the anisotropy factor *g* had a more pronounced effect on absolute fluence values than the scattering coefficient *μ*_*s*_; nevertheless, the spatial distribution and relative light enhancement patterns remained robust across all tested parameter combinations, indicating that qualitative conclusions drawn from the Monte Carlo simulations are not highly sensitive to moderate uncertainty in the retrieved optical properties. Additionally, repeating the analysis with *μ*_*a*_ = 0.001 mm^-1^, representative of dry carbonate minerals, produced minor differences in retrieved *g* (<1.4%) and *μ*_*s*_ (<7%) compared to the water-based value, confirming that the choice of *μ*_*a*_ does not introduce meaningful systematic error.

**Table 2:** Sensitivity analysis of Monte Carlo simulation parameters

Simulation	*μ*_*s*_ [mm^−1^]	*g*	Description	Median surface fluence	Change from baseline (%)
Baseline	11.1	0.96	Median values	3.01	—
Test 1	12.2	0.96	+10% scattering	3.07	+2.0%
Test 2	10.0	0.96	-10% scattering	2.94	-2.3%
Test 3	11.1	0.98	+2% anisotropy	2.57	-14.6%
Test 4	11.1	0.94	-2% anisotropy	3.31	+10.0%

This protocol is broadly applicable to scleractinian coral skeletons and can be adapted to other calcareous, light-dependent taxa. Users should expect variability driven by microscale heterogeneity (necessitating multiple measurements per feature) and occasional measurements that cannot be converted by the lookup grid; these should be reported and filtered rather than forced to boundary values. The sectioned outputs facilitate reproducible reporting, comparative analyses, and integration with physiological or ecological datasets.

## Limitations

This protocol provides quantitative optical characterization of coral microskeletons, but several constraints should be considered when interpreting results. Coral skeletons exhibit microscale variability that can produce spatially heterogeneous measurements; therefore, multiple sampling points per feature are required (n ≥ 5). Additionally, the Monte Carlo simulations assign uniform optical properties to each defined material layer rather than incorporating the full spatial heterogeneity of optical properties observed in the OCT measurements. This simplification is necessary for computational tractability but means the simulations represent average or overall characteristic light behavior. The code does not currently support voxel-by-voxel assignment of different optical properties based on local skeletal features, which would require substantial modification to the workflow. The workflow also isolates skeletal optics and does not reconstruct combined tissue-skeleton interactions that occur in vivo. Light propagation in living corals involves complex interactions between tissue absorption, tissue scattering, and skeletal scattering that cannot be fully reconstructed from separate measurements. Users interested in modeling living coral optics would need to separately characterize tissue optical properties and implement coupled tissue-skeleton models. Finally, this protocol is validated for scleractinian coral skeletons and may require adjustment for other calcareous organisms with different architectures or optical properties.

## Troubleshooting

### Problem 1

Weak OCT signal or elevated background noise (step 10).

### Potential solution

• Ensure the reference arm power indicator is in the “green zone” for optimal signal.
• Confirm the sample surface is clean and dry since residual tissue, dust, or moisture can scatter light and reduce signal quality.
• Adjust the sample to the focal plane based on the reference material: use the air-glass reference if scanning in air, or the water-glass reference if scanning submerged; maximum A-scan intensity indicates proper focus.
• If imaging underwater, minimize water above the sample to reduce signal attenuation.
• Keep ambient lighting consistent (e.g., blinds closed or a darkened room) to avoid fluctuations in background noise.

### Problem 2

Poor exponential fits or unrealistic attenuation/backscatter values (step 17).

### Potential solution

• Visually inspect the A-scan plots (Figure 3) to verify that the surface peak, exponential decay region, and noise floor are correctly identified.
• For highly heterogeneous samples showing multiple distinct decay regions, increase num_slope from 1 to 2 to allow fitting of multiple exponential components. However, avoid overfitting by using more slopes than physically justified by the sample structure.
• Check that calibration constants (a, b, mean_noise) match those obtained from the calibration procedure. Using incorrect calibration parameters will produce systematically wrong *μ* and *ρ* values.

### Problem 3

Experimental data points fall outside the *μ-ρ* lookup grid boundaries (step 18).

### Potential solution

• This commonly occurs for measurements due to measurement noise, calibration uncertainty, or physically extreme optical properties. Filter out NaN values produced during interpolation and focus analysis on the valid data points that fall within the grid.
• Verify that the OCT system parameters (NA, coherence length) used to generate the lookup grid match your actual OCT system specifications. Parameter mismatches will cause systematic shifts in the grid that prevent proper conversion.
• If a large portion (<50%) of measurements fall outside the grid, this may indicate calibration errors. Repeat the full calibration procedure and filter out measurements with poor exponential fit.

### Problem 4

Monte Carlo simulation fails to run or produces “mcxyzn: command not found” error (step 22).

### Potential solution

• Verify mcxyzn executable exists in your MATLAB working directory or the specified path.
• Check that mcxyzn was compiled with OpenMP support. For macOS users, Apple’s default clang compiler does not include OpenMP libraries. Install LLVM via Homebrew, which provides full OpenMP support (see also comment in the top of MC3D.m):

> brew install llvm

> /usr/local/opt/llvm/bin/clang -fopenmp mcxyzn.c -o mcxyzn -lm

• Test the executable directly in terminal: ./mcxyzn should display usage instructions
• Check file permissions to make it executable:

> chmod +x mcxyzn

### Problem 5

Monte Carlo simulation produces unrealistic fluence distributions or fails to complete (step 22).

### Potential solution

• Unit consistency errors are the most common problem. Verify that your STL file dimensions and scattering coefficients use the same units. If your STL is in centimeters but *μ*_*s*_ is in mm^-1^, convert one to match the other.
• If the simulation crashes or runs out of memory, reduce the max_Pxl resolution parameter. Lower resolution reduces memory requirements and computation time but may miss fine skeletal features. Else, try reducing simulation duration.
• Inspect the voxelized geometry by examining the saved label volume and verify that it accurately represents the original STL file. If necessary, smooth or repair the STL file using 3D modeling software before importing.
• If the simulation does not run, verify that the mcxyzn.c has proper permissions and is compatible with your operating system. Check the console output for error messages during execution.

### Problem 6

STL file fails to load or voxelizatoin produces errors (step 22).

### Potential solution

• Verify that the STL file name contains no spaces, parentheses, or special characters. Rename files using underscores instead (e.g., “coral_sample_01.stl”).
• Ensure the STL file is in binary format rather than ASCII format. Most 3D software can export either format; binary files are smaller and load faster.
• Check that the STL file contains a closed, manifold mesh with no holes, self-intersections, or inverted normals. Use 3D mesh repair software to fix mesh errors before importing.
• If the voxelization produces unexpected geometry, inspect the STL file dimensions to ensure they are in reasonable units. Files exported in meters or inches rather than millimeters may cause scaling problems. Convert to millimeters if necessary.
• For very large or complex STL files, voxelization may consume excessive memory. Simplify the mesh by reducing polygon count while preserving essential skeletal features, or reduce the pixel resolution to use coarser voxels.

## Resource availability

### Lead contact

Further information and requests for resources and reagents should be directed to and will be fulfilled by the lead contact, Dr. Daniel Wangpraseurt (dwangpraseurt@ucsd.edu).

### Technical contact

Technical questions on executing this protocol should be directed to and will be answered by the technical contacts, Dr. Netanel Kramer (nati.kramer@gmail.com) for code workflow and execution and Prof. Steven L. Jacques (stevjacq@gmail.com) for optical extraction and Monte Carlo theory.

### Materials availability

This study did not generate new unique reagents.

### Data and code availability

The code and data generated during this study are available at Zenodo: https://doi.org/10.5281/zenodo.19055087.

## Acknowledgments

This work was supported by the Joint United States 10.13039/100000001National Science Foundation (NSF) and 10.13039/100006221United States – Israel Binational Science Foundation (NSF-BSF) grant no. 2021647 to Yossi Loya, no. 2149925 to D.W. and Martin Tresguerres, and in part by funds from the Company of Biologists Fellowship (no. 24051470; sponsored by the Journal of Experimental Biology) to N.K.

## Author contributions

Conceptualization and methodology, N.K., S.L.J., and D.W.; sample collections and lab experiments, N.K.; code writing and analysis, N.K. and S.L.J.; writing – original draft, N.K.; writing – review and editing, N.K., S.L.J., and D.W.; funding acquisition and supervision, D.W.

## Declaration of interests

The authors declare no competing interests.
